# Real-life data on potential drug-drug interactions in patients with chronic hepatitis C viral infection undergoing antiviral therapy with interferon-free DAAs in the PITER Cohort Study

**DOI:** 10.1371/journal.pone.0172159

**Published:** 2017-02-28

**Authors:** Loreta A. Kondili, Giovanni Battista Gaeta, Donatella Ieluzzi, Anna Linda Zignego, Monica Monti, Andrea Gori, Alessandro Soria, Giovanni Raimondo, Roberto Filomia, Alfredo Di Leo, Andrea Iannone, Marco Massari, Romina Corsini, Roberto Gulminetti, Alberto Gatti Comini, Pierluigi Toniutto, Denis Dissegna, Francesco Paolo Russo, Alberto Zanetto, Maria Grazia Rumi, Giuseppina Brancaccio, Elena Danieli, Maurizia Rossana Brunetto, Liliana Elena Weimer, Maria Giovanna Quaranta, Stefano Vella, Massimo Puoti

**Affiliations:** 1 Department of Therapeutic Research and Medicine Evaluation, Istituto Superiore di Sanità, Rome, Italy; 2 Infectious Diseases, Second University of Naples, Naples, Italy; 3 Clinical Unit of Gastroenterology, Azienda Ospedaliera Universitaria Integrata Verona, Verona, Italy; 4 Center for Systemic Manifestations of Hepatitis Viruses (MaSVE), Department of Experimental and Clinical Medicine, University of Florence, Florence, Italy; 5 Department of Infectious Diseases, San Gerardo Hospital, University of Milano-Bicocca, Monza, Italy; 6 Department of Internal Medicine, University Hospital of Messina, Messina Italy; 7 Section of Gastroenterology, Department of Emergency and Organ Transplantation, University of Bari, Bari, Italy; 8 Infectious Diseases, Azienda Ospedaliera Santa Maria Nuova di Reggio Emilia, Reggio Emilia, Italy; 9 Infectious Diseases, University of Pavia, Pavia, Italy; 10 Gastroenterology, Department of Surgical and Gastroenterological Sciences, University of Udine, Udine, Italy; 11 Department of Surgical and Gastroenterological Sciences, University Hospital of Padua, Padua, Italy; 12 Division of Hepatology, Ospedale San Giuseppe, Università degli Studi di Milano, Milan, Italy; 13 Division of Infectious Diseases, Azienda Ospedaliera Ospedale Niguarda Ca' Granda, Milan, Italy; 14 Hepatology Unit, University Hospital of Pisa, Pisa, Italy; University of North Carolina at Chapel Hill School of Dentistry, UNITED STATES

## Abstract

**Background:**

There are few real-life data on the potential drug-drug interactions (DDIs) between anti-HCV direct-acting antivirals (DAAs) and the comedications used.

**Aim:**

To assess the potential DDIs of DAAs in HCV-infected outpatients, according to the severity of liver disease and comedication used in a prospective multicentric study.

**Methods:**

Data from patients in 15 clinical centers who had started a DAA regimen and were receiving comedications during March 2015 to March 2016 were prospectively evaluated. The DDIs for each regimen and comedication were assigned according to HepC Drug Interactions (www.hep-druginteractions.org).

**Results:**

Of the 449 patients evaluated, 86 had mild liver disease and 363 had moderate-to-severe disease. The use of a single comedication was more frequent among patients with mild liver disease (p = 0.03), whereas utilization of more than three drugs among those with moderate-to-severe disease (p = 0.05). Of the 142 comedications used in 86 patients with mild disease, 27 (20%) may require dose adjustment/closer monitoring, none was contraindicated. Of the 322 comedications used in 363 patients with moderate-to-severe liver disease, 82 (25%) were classified with potential DDIs that required only monitoring and dose adjustments; 10 (3%) were contraindicated in severe liver disease. In patients with mild liver disease 30% (26/86) used at least one drug with a potential DDI whereas of the 363 patients with moderate-to-severe liver disease, 161 (44%) were at risk for one or more DDI.

**Conclusions:**

Based on these results, we can estimate that 30–44% of patients undergoing DAA and taking comedications are at risk of a clinically significant DDI. This data indicates the need for increased awareness of potential DDI during DAA therapy, especially in patients with moderate-to-severe liver disease. For several drugs, the recommendation related to the DDI changes from “dose adjustment/closer monitoring”, in mild to moderate liver disease, to “the use is contraindicated” in severe liver disease.

## Introduction

The new generation of oral direct-acting antivirals (DAAs) has transformed the treatment of hepatitis C virus (HCV) infection, demonstrating both high efficacy and high tolerability [1–3]. However, none of the DAAs are completely free of drug-drug interactions (DDIs), which can significantly alter the drugs’ exposure and thus their efficacy and toxicity. Studies on interactions between DAAs and some key drugs have been performed in the development of all DAAs. However, the majority of clinical trial participants have been healthy volunteers with few comorbidities and limited concomitant medications [4,5].

Clinical implications of established or potential DDIs between DAAs and comedications vary, as do the effects of hepatic and renal impairment on DAAs and other drugs. Interactions may lead to decreased concentrations resulting in decreased efficacy (i.e lack of therapeutic effect) or increased peak concentrations associated with increased drug toxicity.

In patients with severe liver disease, determining the effect of DDIs between DAAs and comedications remains a challenge. This challenge is further complicated by ageing and additional comorbidities in chronic HCV patients, often resulting in polypharmacy.

There is limited data available that addresses DDIs in patients with chronic HCV infection [6]. The objective of the present study was to assess the potential DDIs of DAAs with medications used in outpatients that began anti-HCV Interferon (IFN)-free therapy as part of the PITER Cohort Study (Piattaforma Italiana per lo studio della Terapia delle Epatiti viRali) [7].

## Patients and methods

### Patients

For the present prospective multicentric real life study, we evaluated data from patients who were initiated a DAA IFN-free regimen in the period from March 2015 to March 2016 and who were receiving at least one comedication. The data were provided by those 15 clinical centers involved in the PITER Cohort Study that had available data on comedication during DAA therapy. PITER is a collaboration involving Italy’s National Institute of Public Health (Istituto Superiore di Sanità), the Italian Society for the Study of the Liver (AISF), and the Italian Society for Infectious Diseases (SIMIT) [7]. The data were collected prospectively from the prescribing clinician when the regimen was started, on the electronic case-report form used for PITER. The DAAs available during the time period of this study which were evaluated in the present work are reported in Table 1.

**Table 1:** Sociodemographic and virological characteristics and comedications used, by severity of liver disease, among HCV-infected patients undergoing DAA therapy.

Patient Characteristics	Severity of Liver Disease		p-value
	Mild N. Patients: 86 (%)	Moderate-to-Severe N. Patients: 363 (%)	
Median age	64 years (range: 29–82)	65 years (range: 45–82)	0.7
Gender: male/female	38/48 (44/54)	217/145 (60/40)	<0.01
**Genotype Distribution**			
1a	9 (11)	48 (13)	0.6
1b	46 (53)	186 (51)	0.7
2	17 (20)	52 (14)	0.2
3	7 (8)	55 (15)	0.1
4	7 (8)	22 (6)	0.5
**Comedications**			
1 drug	34 (40)	100 (28)	0.03
2 drugs	18 (21)	81 (22)	0.9
3 drugs	15 (17)	65 (18)	1
4 drugs	10 (12)	55 (15)	0.4
5 drugs	5 (6)	32 (9)	0.5
>5 drugs (range 6–12)	4 (5)	33 (9)	0.2
**DAA regimens**			
Sofosbuvir+Ribavirin	28 (33)	105 (29)	0.5
Sofosbuvir+Simeprevir	20 (23)	95 (26)	0.7
Sofosbuvir+Daclatasvir	6 (7)	40 (11)	0.3
Sofosbuvir+Ledipasvir	9 (10)	44 (12)	0.8
Paritaprevir/ritonavir, ombitasvir, dasabuvir	23 (27)	78 (21)	0.3

We evaluated DDIs according to the severity of liver disease and the specific comedications used. The fibrosis stage was defined based on liver transient elastometry data, which were considered validated if each patient had at least 10 valid stiffness measurements, with a success rate of at least 80%, an interquartile range of less than 30% of the median stiffness score, and a Body Mass Index (BMI) of <30kg/m^2^. The severity of liver disease was classified as “mild” if the stiffness score was equal to or lower than 10 kPa and as “moderate-to-severe” if it was higher or if there were signs of liver cirrhosis (signs of portal hypertension) [8]. Patients coinfected with HIV or HBV and those included in clinical trials were excluded.

### Assessment of comedications

Potential DDIs were assessed and classified based on information available at www.hep-druginteractions.org. For most interactions, the information was based on the metabolism pathway of each drug used, in the absence of clinical data. Specifically, the DDIs for each DAA regimen and each drug used as comedication were assigned to four different risk categories:

Category 0: Classification not possible due to lack of information
Category 1: No clinical interaction possible
Category 2: May require dose adjustment/closer monitoring
Category 3: Co-administration not recommended or contraindicated

Each DDI was evaluated considering the stage of liver disease of each patient. Safety concerns for a comedication due to hepatic impairment and not only due to an interaction with the DAA were also considered in this study. Specifically, some DDIs were considered as Category 2 or Category 3 only in patients with moderate-to-severe liver disease, whereas they were considered as Category 1 in patients with mild liver disease.

### Statistical analysis

Differences among the proportions were evaluated by chi-square or Fisher test, as appropriate. A p-value of less than 0.05 was considered as significant.

### Ethics

The protocol of PITER was approved by the Ethics Committee of ISS on 19th June 2013, and by the Ethics Committees of each participating institution that are listed in the PITER Collaborating Group available at www.iss.it/piter. All patients included in the database signed an informed consent prior to enrolment. The patients’ data were evaluated through an anonymous analysis, adopting codes generated by the electronic case-report form.

## Results

### Characteristics of the study population and medications used

Of 147 patients who had mild disease (median stiffness: 8.1kPa; range: 4.1–9.9) and of 550 patients who had moderate-to-severe liver fibrosis and/or cirrhosis (median stiffness: 20.8; range: 10.5–68.1; or clinical signs of liver cirrhosis), 86 (58%) and 363 (66%) patients respectively were receiving comedications respectively. The characteristics of the patients and information on comedications, by severity of liver disease at the time of starting DDA treatment, are reported in Table 1.

The use of a single comedication was more frequent among patients with mild liver disease (p = 0.03), whereas the use of more than three drugs was reported in 19 (22%) and 120 (33%) patients with mild and moderate-to-severe liver disease respectively (p = 0.05). The most frequently reported DAA regimens were sofosbuvir plus ribavirin (SOF/RBV), followed by paritaprevir/ritonavir, ombitasvir, dasabuvir (referred to as 3D) and sofosbuvir plus simeprevir (SOF/SIM). The least common used regimens were sofosbuvir plus ledipasvir (SOF/LDV) and sofosbuvir plus daclatasvir (SOF/DCV). There were no significant differences in use between patients with mild compared to those with moderate to severe liver disease. In patients with moderate-to-severe disease, the 3D regimen was used only in patients with Child-Pugh A cirrhosis.

The drug classes of the comedications are reported in Table 2. Proton pump inhibitors, diuretics and beta blockers were more frequently used in patients with moderate-to-severe liver disease, whereas anxiolytic drugs were more common in patients with mild liver disease. Similar frequencies were reported for other drug classes. Antipsychotic/antidepressives drugs used in both populations are mainly selective serotonin reuptake inhibitor of which only sertraline is reported with possible DDIs.

**Table 2:** Drug classes used as comedication when beginning DAA therapy, by severity of liver disease, among HCV-infected patients.

Drug Class*	Severity of Liver Disease		p-value
	Mild	Moderate-to-Severe	
	N. Patients: 86 (%)	N. Patients: 363 (%)	
Ace Inhibitors	11 (13)	65 (18)	0.2
Antipsychotic/Antidepressives	12 (14)	40 (11)	0.4
Antiaggregant/Anticoagulant	15 (17)	48 (13)	0.3
Antibacterials/antiprotozoal	7 (8)	19 (5)	0.3
Antidiabetics	15 (17)	71 (20)	0.6
Anxyolitic/Hypnotic/Sedatives/	19 (22)	43 (12)	0.01
Beta blockers	17 (20)	126 (35)	<0.01
Ca antagonists	9 (11)	27 (7)	0.3
Diuretic (component of antihypertensive therapy)	15 (18)	155 (43)	<0.001
Gastrointestinal other than PPI	10 (12)	60 (17)	0.2
PPI	16 (19)	124 (34)	<0.01
Sartanic	15 (17)	52 (14)	0.4
Substitute Hormonal therapy	11 (13)	30 (8)	0.2

*Reported drug classes used in more than 5% of each group.

**Fig 1:**
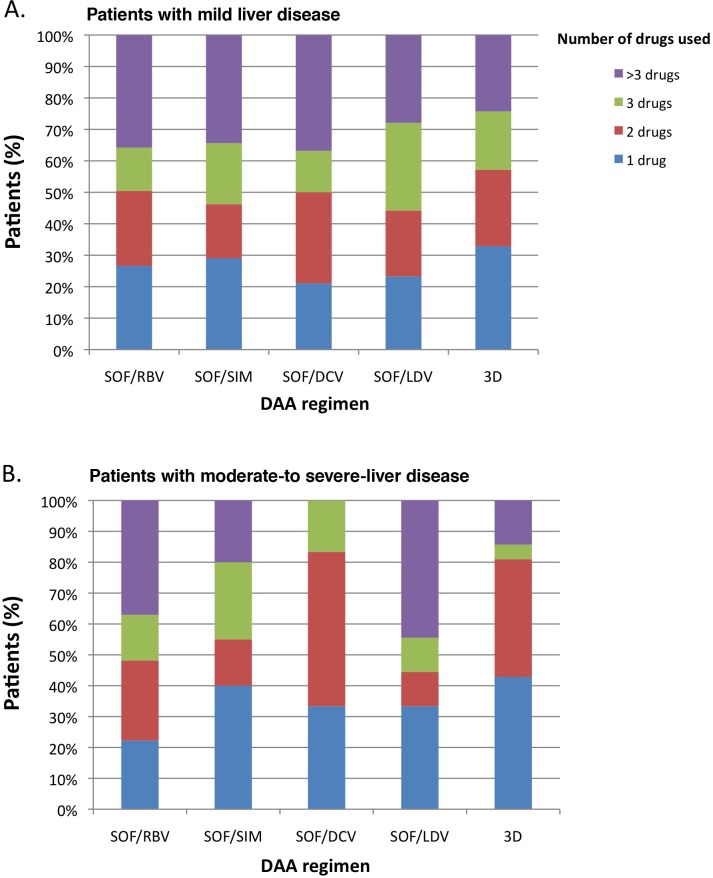
Number of co-medications used and percentage of patients, by DAA regimen, among HCV-infected patients. (A) Patients with mild liver disease. (B) Patients with moderate-to severe-liver disease. SOF/RBV: sofosbuvir plus ribavirin, SOF/SIM: sofosbuvir plus simeprevir, SOF/DCV: sofosbuvir plus daclatasvir, SOF/LDV: sofosbuvir plus ledipasvir, 3D: paritaprevir/ritonavir, ombitasvir, dasabuvir. The percentage of patients who took one drug (in blu), two drugs (in red), three drugs (in green) and more than 3 drugs (in violet) are reported considering the total number of patients reported for each regimen in both Fig 1A and Fig 1B at the same manner.

The number of comedications, by DAA regimen, is reported in Fig 1A for patients with mild disease and in Fig 1B for patients with moderate-to-severe disease.

### Comedications with potential DDIs

**Fig 2:**
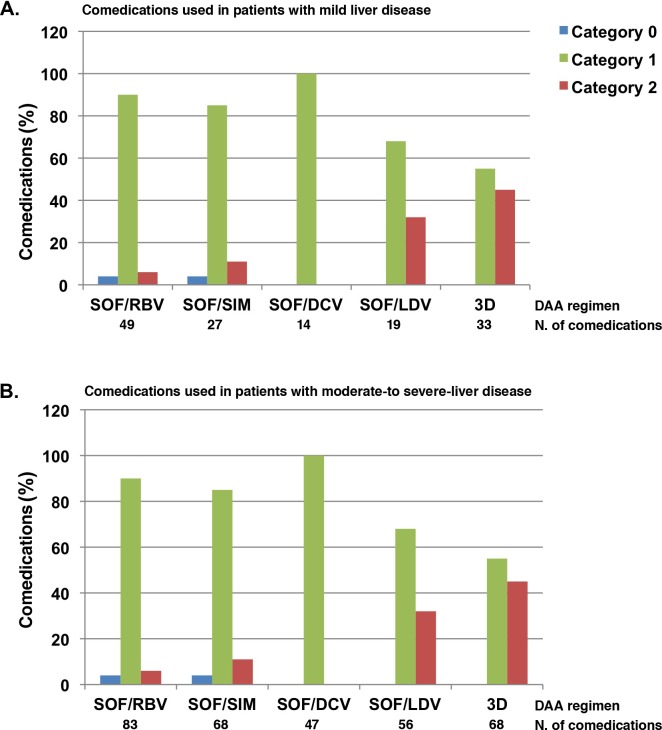
Category of potential DDIs, by DAA regimen and severity of liver disease, among HCV-infected patients. Comedication used in patients with mild liver disease (A) or in (B) patients with moderate-to severe-liver disease (B). DAA regiments and number of comedications used are shown. SOF/RBV: sofosbuvir plus ribavirin, SOF/SIM: sofosbuvir plus simeprevir, SOF/DCV: sofosbuvir plus daclatasvir, SOF/LDV: sofosbuvir plus ledipasvir, 3D: paritaprevir/ritonavir, ombitasvir, dasabuvir. Category 0: Classification not possible due to lack of information; Category 1: No clinical interaction possible; Category 2: May require dose adjustment/closer monitoring.

The classification of DDIs, by severity of liver disease, is reported in Fig 2.

In both groups of patients, the greatest percentage of DAAs regimens with potential DDIs was reported for the 3D regimen (45% of drugs used in patients with mild liver disease and 38% of drugs used in those with moderate-to-severe liver disease). In (80%) (12/15) of drugs used in patients with mild liver disease and 54% (14/ 26) of those used in patients with moderate-to-severe liver disease in combination with the 3D regimen, a DDI was reported between ritonavir component of the 3D regimen and each drug used as comedication.

Overall, of the 142 comedications prescribed in patients with mild liver disease, 27 (20%) were classified as Category 2 (“May require dose adjustment/closer monitoring”).

Overall, of the 322 comedications used in patients with moderate-to-severe liver disease, 82 drugs (25%) were classified as Category 2; of these, 10 (3%) were classified as Category 3 in cases of their use in severe liver disease. All patients in whom these drugs were co administered were in the Child-Pugh A liver cirrhosis stage.

The drugs most commonly reported as having at least one DDI in patients with moderate-to-severe liver disease are shown in Table 3.

**Table 3:** The most common drugs with a potential DDI among HCV-infected patients with moderate-to-severe liver disease.

Drug	N. Patients (%)	DAA Regiments with Possible Category 2 DDIs
PPI	124 (34.2)	SOF/LDV; 3D
Propranolol	70 (19.5)	3D
Furosemide	56 (15.4)	3D
Levothyroxin	30 (8.2)	SOF/DCV; 3D
Lactulose	26 (7.1)	3D
UDCA	26 (7.1)	SOF; SOF/SIM; SOF/DCV; SOF/LDV
Amlodipin	24 (6.6)	SOF/SIM; SOF/DCV; SOF/LDV; 3D
Carvedilol*§	18 (5)	SOF; SOF/SIM; SOF/DCV; SOF/LDV; 3D
Rifaximin	18 (4.9)	3D
Irbesartan	15 (4.1)	SOF/LDV; 3D
Prednisone	14 (3.8)	SOF/SIM; 3D
Alpraxolam	12 (3.3)	3D
Olmesartan*§	12 (3.3)	SOF; SOF/SIM; SOF/LDV; 3D
Bisoprolol	11 (3)	SOF; SOF/SIM; SOF/LDV; 3D
Sertralin§	8 (2.2)	SOF; SOF/SIM; SOF/DCV; 3D
Telmisartan	8 (2.2)	3D
Allopurinolo	7 (1.9)	SOF; SOF/SIM; SOF/DCV; SOF/LDV
Doxazosin§	7 (1.9)	SOF; SOF/SIM; SOF/LDV; 3D
Enalapril	7 (1.9)	3D
Valsartan	7 (1.9)	SOF; SOF/SIM; SOF/DCV; 3D
Nebivolol§	6 (1.6)	SOF/SIM; SOF/DCV; SOF/LDV
Propafenone	6 (1.7)	SOF/SIM; SOF/LDV; 3D
Candesartan§	5 (1.1)	SOF; SOF/LDV; 3D
Lisinopril	5 (1.1)	SOF/SIM; SOF/LDV
Lormetazepam	5 (1.1)	3D

*Dose adjustment only in the European Summary Product Characteristics. No dosage restrictions in the US prescribing Information.

§ Contraindicated in patients with severe hepatic impairment.

### Use of comedications with potential DDIs in the study population

Of the 86 patients with mild liver disease, 26 (30%) used at least one drug with a potential DDI (Category 2). Of these 26 patients, 11 (42%) were treated with the 3D regimen, the remaining 15 patients were equally distributed among the other regimens.

None of patients with mild liver disease used a contraindicated drug (Category 3) as comedication.

Of the 363 patients with moderate-to-severe liver disease, 161 (44%) had more than one DDI. Potential DDIs were more frequently found for the 3D regimen (n = 48 patients, 36%), followed by SOF/SIM (n = 40 patients, 25%).Similar prevalence rates of patients at risk for at least one DDI were observed for the remaining sofosbuvir-based regimens (data not shown). The 10 drugs reported to be contraindicated in severe liver disease were used by 63 (17%) patients in with similar prevalence for all of the DAA regimens. No important clinical outcomes were reported for these patients during the study period. However, this study is prospective in design and the evaluation of important clinical outcomes and of the potential DDIs is ongoing.

## Discussion

Polypharmacy has become an important issue among patients with HCV mono-infection, and DDIs are one of the challenges in the DAA-based treatment of these patients [9,10]. The most frequently reported drug interactions modify drug metabolism by inducing or inhibiting the cytochrome P450, leading to abnormal drug exposure [10].

Many of the DDI studies have been performed in healthy volunteers, yet HCV-infected patients with cirrhosis may have impaired CYP450 capacity and higher plasma concentrations of CYP450 substrates compared to healthy individuals. This would mean that they are at even more risk for drug toxicity when a DDI occurs. In light of this, different profiles of potential drug-drug interactions have been hypothesized in patients with moderate-to-severe liver disease, however, few data are available for real-life patients [11–13].

Our real-life data stress that potential DDIs are an important clinical issue for individuals treated with DAAs for chronic HCV infection. We found that a wide variety of drugs belonging to different classes were used, even wider than that reported by Siederdissen *et al*. [6], who conducted a single center survey and whose patients were around 10 years younger, presumably with fewer comorbidities than those in our cohort.

The profile of the patients in our study mirrored the epidemiology of HCV infection in Italy, whose prevalence is greatest among the elderly [14]. As a consequence, in our cohort, polypharmacy was relatively common in patients with mild liver disease as in those with moderate-to-severe liver disease. Of the patients with mild liver disease, 30% reported a potential Category 2 DDI, for which the most suitable approach is monitoring for the early detection of adverse events [6,15]. These data indicate that in patients with mild liver disease, through careful pre-treatment assessment of concomitant medications and monitoring or dose-modifications, significant DDIs can be avoided even in elderly patients who generally take multiple comedications for different comorbidities [10,16–19]. However, the use of contraindicated comedications (Category 3 of DDI) should always be checked and, if present, an alternative comedication should be provided, regardless of the severity of liver impairment. Our data showed that none of the patients with mild liver disease were taking contraindicated comedications during DAA treatment, whereas 10% of the comedications were contraindicated in patients with moderate-to-severe liver disease. Patients with moderate-to-severe liver disease were a group of particular interest, due to the intersection between older age, comorbidities and severity of liver disease. In this study, 44% of patients with severe liver disease were affected by more than one DDI. Of these patients, 17% used comedications that are contraindicated in cases of severe liver damage, mainly because of the possible deterioration of liver disease. That these drugs were prescribed and the lack of important clinical outcomes during ongoing DAA therapy could be explained by the fact that all were classified with Child-Pugh A liver cirrhosis, which indicated that the liver impairment was not very severe. However, clinicians should be aware of the possible interactions reported for different comedications and DAAs, in particular in patients with severe liver impairment [20].

Our series showed that DAA regimens containing a protease inhibitor (3D combination with ritonavir or SOF/SIM) was associated with a higher risk for DDIs (38% and 32%, respectively), compared to other SOF-containing regimens (11–23%). Furthermore, these regimens were contraindicated in patients with advanced/decompensated liver cirrhosis. The mechanism of DDIs in patients receiving the 3D regimen can primarily be attributed to the ritonavir component of 3D, whose mechanism of action is to modify the metabolism of concomitant drugs, mainly increasing concomitant drug concentrations [16].

Warnings on the administration of comedications with the DAA regimens that include protease inhibitors (3D and Simeprevir regimens) were released in 2015, when our data were being collected, which, over time, may have increased the awareness of possible DDIs related to these regimens [15,21,22].

In general, regimens with the NS5B inhibitor sofosbuvir plus an HCV NS5A inhibitor (i.e., ledipasvir, daclatasvir), which do not affect CYP450, were relatively free of significant pharmacokinetic interactions, even in patients with moderate to severe liver impairment.

PPIs were the most frequently used comedication in our study (used in 19% and 34% of patients with mild and moderate-to-severe liver disease, respectively). The possible DDIs between PPIs and DAAs has been emphasized recently, given that gastric pH could affect DAA bioavailability due to increased or decreased pharmacokinetics, as reported for 3D and SOF/LDV and in other DAA regimens containing NS3/4A protease inhibitors, such as grazoprevir, and the NS5A inhibitor elbasvir [23–26]. However, the finding of a post-hoc analysis provides reassurance that the co-administration of 3D and PPI does not negatively affect the chance of viral eradication [27].

In conclusion, hundreds of thousands of patients are currently being treated with DAAs, and, based on our real-life data, 30–44% of those taking comedications are at risk of a DDI. For several drugs, the recommendation related to a potential DDI depends on the severity of liver disease, and a careful evaluation of DDIs is required, particularly in patients with severe liver impairment. This stresses the need for increased awareness of this issue and for additional extensive research.
