# Correction: Real-life data on potential drug-drug interactions in patients with chronic hepatitis C viral infection undergoing antiviral therapy with interferon-free DAAs in the PITER Cohort Study

**DOI:** 10.1371/journal.pone.0190803

**Published:** 2018-01-02

**Authors:** Loreta A. Kondili, Giovanni Battista Gaeta, Donatella Ieluzzi, Anna Linda Zignego, Monica Monti, Andrea Gori, Alessandro Soria, Giovanni Raimondo, Roberto Filomia, Alfredo Di Leo, Andrea Iannone, Marco Massari, Romina Corsini, Roberto Gulminetti, Alberto Gatti Comini, Pierluigi Toniutto, Denis Dissegna, Francesco Paolo Russo, Alberto Zanetto, Maria Grazia Rumi, Giuseppina Brancaccio, Elena Danieli, Maurizia Rossana Brunetto, Liliana Elena Weimer, Maria Giovanna Quaranta, Stefano Vella, Massimo Puoti

The graph that appears as Fig 2A is incorrectly duplicated in Fig 2B. Please see the corrected Fig 2 here.

**Fig 2:**
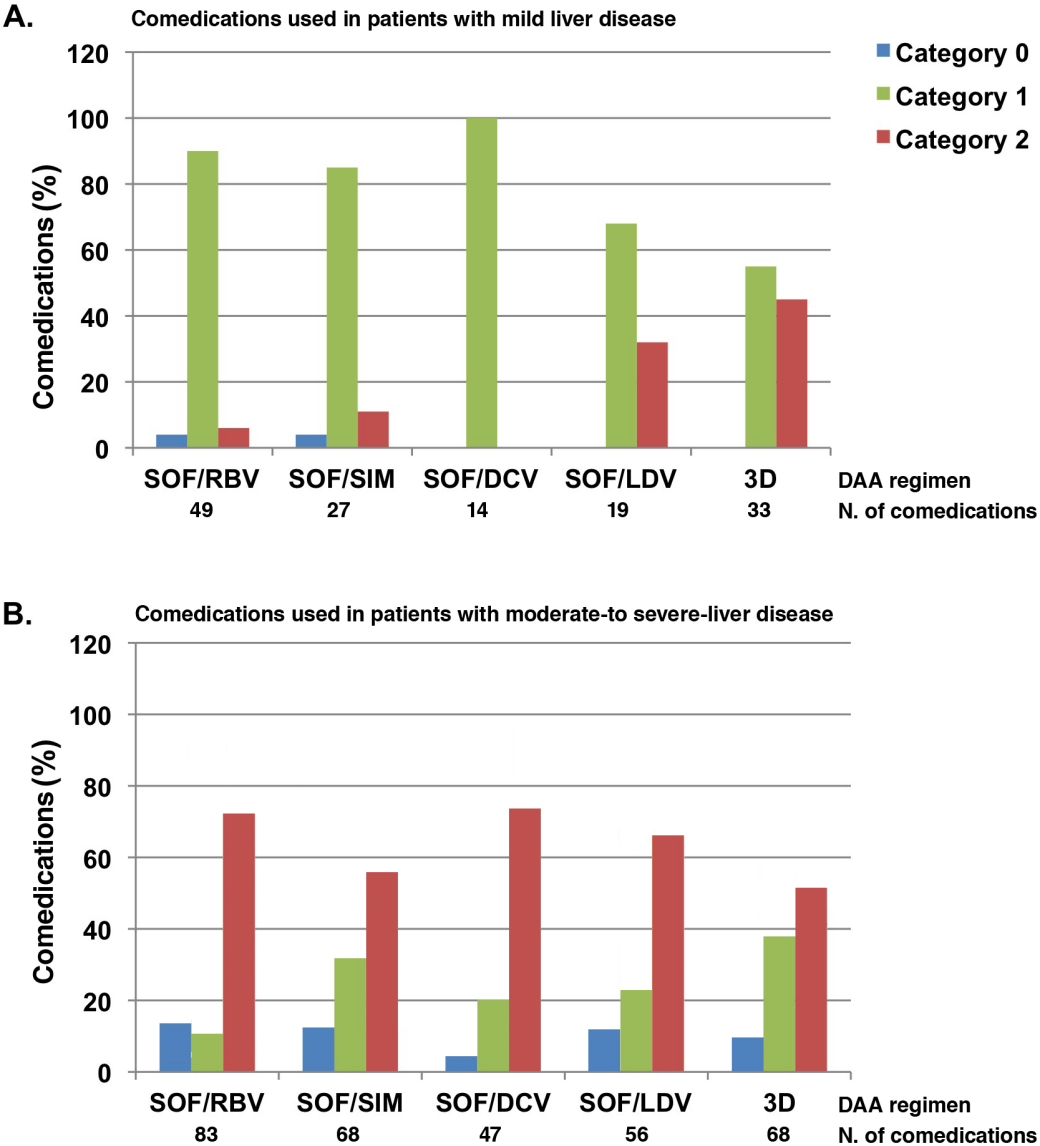
Category of potential DDIs, by DAA regimen and severity of liver disease, among HCV-infected patients. Comedication used in patients with mild liver disease (A) or in (B) patients with moderate-to severe-liver disease (B). DAA regiments and number of comedications used are shown. SOF/RBV: sofosbuvir plus ribavirin, SOF/SIM: sofosbuvir plus simeprevir, SOF/DCV: sofosbuvir plus daclatasvir, SOF/LDV: sofosbuvir plus ledipasvir, 3D: paritaprevir/ritonavir, ombitasvir, dasabuvir. Category 0: Classification not possible due to lack of information; Category 1: No clinical interaction possible; Category 2: May require dose adjustment/closer monitoring.
